# CRBN Is a Negative Regulator of Bactericidal Activity and Autophagy Activation Through Inhibiting the Ubiquitination of ECSIT and BECN1

**DOI:** 10.3389/fimmu.2019.02203

**Published:** 2019-09-18

**Authors:** Mi-Jeong Kim, Yoon Min, Jae-Hyuck Shim, Eunyoung Chun, Ki-Young Lee

**Affiliations:** ^1^Department of Molecular Cell Biology and Samsung Biomedical Research Institute, Sungkyunkwan University School of Medicine, Suwon, South Korea; ^2^Division of Rheumatology, Department of Medicine, University of Massachusetts Medical School, Worcester, MA, United States; ^3^Department of Immunology and Infectious Diseases, Harvard School of Public Health, Boston, MA, United States; ^4^Department of Medicine, Harvard Medical School, Boston, MA, United States; ^5^Department of Health Sciences and Technology, Samsung Medical Center, Samsung Advanced Institute for Health Sciences and Technology, Sungkyunkwan University, Seoul, South Korea

**Keywords:** cereblon, toll-like receptor 4, TRAF6, ECSIT, BECN1, ubiquitination, mROS

## Abstract

Cereblon (CRBN) as a multifunctional protein has been extensively studied. Here, we show that CRBN is a negative regulator of bactericidal activity and autophagy activation. Mitochondrial localization of CRBN was significantly increased in response to Toll-like receptor 4 (TLR4) stimulation. CRBN interrupted the association of evolutionarily conserved signaling intermediate in Toll pathways (ECSIT)-TNF-receptor associated factor 6 (TRAF6) complex, thereby inhibiting the ubiquitination of ECSIT, which plays a pivotal role for the production of mitochondrial reactive oxygen species (mROS). Subsequently, mROS levels were markedly elevated in CRBN-knockdown (CRBN^KD^) THP-1 cells, and that led to resistance against S. typhimurium infection, indicating CRBN is a negative regulator of bactericidal activity through the regulation of mROS. Additionally, CRBN inhibited TRAF6-induced ubiquitination of BECN1 (Beclin 1), and that induced autophagy activation in CRBN^KD^ THP-1, CRBN-knockout (CRBN^KO^) H1299, and CRBN^KO^ MCF7 cancer cells in response to TLR4 stimulation. Notably, we found that the ability of cancer migration and invasion was significantly enhanced in CRBN^KO^ H1299 and CRBN^KO^ MCF7 cancer cells, as compared with those of control cancer cells. Collectively, these results suggest that CRBN is a negative regulator of bactericidal activity and autophagy activation through inhibiting the TRAF6-induced ubiquitination of ECSIT and BECN1, respectively.

## Introduction

Cereblon (CRBN) was known to be a candidate gene that causes autosomal recessive non-syndromic mental retardation (1, 2). CRBN is distributed in subcellular compartments, including the nucleus, cytoplasm, and endoplasmic reticulum, and the peripheral membrane of the human brain and other tissues (3–5). Subsequently, a comprehensive study has extensively investigated the various functions of CRBN (1–7). CRBN as a component of the Cullin 4-RING E3 ubiquitin ligase complex is known to be a target of thalidomide (4). The association of CRBN and thalidomide initiates teratogenic effects through the inhibition of the associated ubiquitin ligase activity (4). Additionally, CRBN is associated with thalidomide and its analogs to induce proteasome-dependent degradation of IKZF1 and IKZF3 transcription factors, leading to the growth inhibition, and apoptosis of multiple myeloma (MM) cells, following downregulation of c-Myc and IRF4 (8–12). Moreover, CRBN interacts with the cytoplasmic region of large-conductance calcium-activated potassium channels (13), voltage-gated chloride channel-2 (ClC-2) (14), and the α1 subunits of AMP-activated protein kinase (AMPK) (15). These interactions critically affect the functional roles of target proteins through the regulation of surface expression, cellular assembly, and kinase activity, respectively (13–15). A recent report has shown that CRBN distributes in mitochondria and has a protective function against oxidative stress, similar to Lon protease, suggesting that mitochondrial CRBN is critically implicated in cytotoxicity induced by oxidative stress (16). However, whether CRBN is implicated in the production of mitochondrial reactive oxygen species (mROS), and is thereby functionally involved in the cellular roles of mROS, has not yet been investigated.

Mitochondrial reactive oxygen species as reactive oxygen species are produced by mitochondria, and functionally contribute to bactericidal activity in innate phagocytic cells (17–20). However, the molecular and cellular mechanism by which mROS production is implicated in these processes remains unclear. Recent reports have suggested that signals of Toll-like receptors (TLRs) are critically involved in the effective phagosome-mitochondrion function and bactericidal activity (19, 20). Mst1 and Mst2 kinases induce a molecular association between TRAF6 and ECSIT following TLR stimulation, which have been shown to regulate bactericidal activity by regulating ROS production (20). More importantly, the translocation of mitochondria to phagosomes by TLR engagement in macrophages was found to be regulated by the TRAF6-ECSIT complex (19, 20). The association induced ubiquitination of ECSIT by TRAF6 and the augmentation of mROS production, thereby leading to bactericidal activity, indicating the pivotal role of ubiquitin ligase activity of TRAF6 in mROS production (19, 20). Notably, recent reports have shown that the ubiquitin-ligase activity of TRAF6 plays an important role in the initiation and function of autophagy (21–23). TRAF6 regulated TLR4-induced autophagy via the ubiquitination of BECN1 (Beclin 1) (21, 23). Additionally, activation of TLR4 and TLR3 stimulated autophagy induction in lung cancer cells and induced enhancements of cytokine productions, such as IL-6, CCL2/MCP-1, CCL20/MIP-3α, VEGFA, and MMP2, by encouraging TRAF6 ubiquitination, thereby led to increases of cell migration and invasion (22). These results strongly suggest that TRAF6 as a pivotal regulator of TLRs-mediated signals might play a key role in regulating mROS production and autophagy activation in a ubiqutin ligase dependent manner.

Here, we provide insight into the functional roles of CRBN in bactericidal activity and autophagy activation. CRBN inhibits the ubiquitination of ECSIT, and of BECN1, by TRAF6. CRBN-knockdown (CRBN^KD^) THP-1 cells revealed increases of mROS levels and resistance against S. typhimurium infection. Moreover, autophagy activation was markedly enhanced in CRBN^KD^ THP-1, CRBN-knockout (CRBN^KO^) H1299 and CRBN^KO^ MCF7 cells, in response to TLR4 stimulation. Interestingly, cancer migration and invasion ability were significantly enhanced in CRBN^KO^ H1299 and CRBN^KO^ MCF7 cancer cells. Together, our results provide evidence, and the underlying molecular mechanism that CRBN is a negative regulator of bactericidal activity and autophagy activation, through the inhibition of TRAF6 ubiquitin ligase activity.

## Materials and Methods

### Antibodies and Reagents

#### Antibodies

Anti-CRBN (Ca# ab68763), anti-GRIM19 (Ca# ab134325), and anti-ECSIT (Ca# ab21288) were purchased from Abcam, Cambridge, MA, USA. Anti-TRAF6 (Ca# sc-7221) was purchased from Santa Cruz Biotechnology, Dallas, Texas, USA. Anti-LC3A/B (Ca# 4108S), anti-GAPDH (Ca# 2118S), anti-Myc (Ca# 2276), and anti-IκB (Ca# 9242) were purchased from Cell Signaling Technology, Danvers, MA, USA. Anti-Flag (Ca# F3165), and anti-HA (Ca# H3663) were purchased from Sigma-Aldrich, St. Louis, MO, USA.

#### Reagent Materials

4′,6-Diamidine-2′-phenylindole dihydrochloride (DAPI), lipopolysaccharide (LPS, Ca#L2887), 3-Methyladenine [3-MA, Ca# M9281], Chloroquine (CQ, Ca# C6628), dimethyl sulfoxide (DMSO, Ca# 472301), puromycin (Ca# P9620), paraformaldehyde (Ca# P6148), Triton X-100 (Ca# T9284), phorbol 12-myristate13-acetate (PMA, Ca# 16561298), gentamicin (Ca# G1272), deoxycholate (Ca# D6750), and Dulbecco'sphosphate-buffered saline (DPBS, Ca# D8537) were purchased from Sigma-Aldrich (St. Louis, MO, USA). MitoTracker Red (Ca# M7512) and Lipofectamine 2000 (Ca# 11668-019) were purchased from Thermo Scientific, Rockford, IL, USA. MitoSOX Red (Ca# M36008) was purchased from Molecular Probes, Invitrogen, Carlsbad, CA, USA.

### Plasmid Constructs

#### Plasmids

Flag-tagged TRAF6 (Ca# 21624), Flag-tagged CRBN (Ca# 107380), and Flag-tagged BECN1 (Ca# 24388) were purchased from Addgene, Cambridge, MA, USA. Expressing vectors, such as Myc-tagged ECSIT, HA-tagged Ub, and Flag-tagged ECSIT, were obtained from Jae-Hyuck Shim (University of Massachusetts Medical School, USA). Full-length TRAF6 and CRBN were cloned into the pCMV-3Tag-7 vector (Ca# 240202, Agilent technologies) using Flag-tagged TRAF6 and Flag-tagged CRBN, respectively, as a template. Myc-tagged CRBN were used as vector backbone to generate Myc-tagged CRBN 1-261 and Myc-tagged CRBN 1-186 truncated mutants using the primers: Myc-tagged CRBN 1-261: forward primer, 5′-ATAGGATCCATGGCCGGCGAAGGAGAT-3′; reverse primer, 5′-GGCCTCGAGTCATAGCTGTTTCTTGATTCTGTC-3′; Myc-tagged CRBN 1-186: forward primer, 5′-ATAGGATCCATGGCCGGCGAAGGAGAT-3′; reverse primer, 5′-GGCCTCGAGCTAGGGAAGAATTTGCACTTT-3′. Flag-tagged ECSIT truncated mutants were generated as previously described (24).

### Cell Culture

HEK293T human embryonic kidney cells (Ca# CRL-11268, ATCC, Manassas, VA, USA) were cultured and maintained in Dulbecco's modified Eagle's medium (DMEM; CA#11965092, Thermo Fisher Scientific). THP-1 human monocytic cells (Ca# TIB-202, ATCC) were cultured and maintained in RPMI 1640 medium (Ca#11875093, Thermo Fisher Scientific) with 10% fetal bovine serum (FBS; Fisher Scientific Hyclone, Ca#11306060), 2 mM L-glutamine (GIBCO, Ca#A2916801), 100 units/ml penicillin (GIBCO, Ca#15140122), 100 μg/ml streptomycin (GIBCO, Ca#15140122), and 5 × 10^−5^ M β-mercaptoethanol (GIBCO, Ca#21985023). Human non-small cell lung carcinoma cell line H1299 (Ca# CRL-5803) and human breast adenocarcinoma cell line MCF7 cells (Ca# HTB22) were obtained from ATCC, and maintained in DMEM supplemented with 10% FBS.

### Generation of CRBN-Knockdown Cell Line

Lentivirus containing small hairpin RNA (shRNA) targeting human CRBN (Ca# sc-78528-V) or control shRNA lentivirus (Ca# sc-108080) were purchased from Santa Cruz Biotechnology (Santa Cruz, CA, USA). THP-1 cells were cultured in wells of a 24-well plate (2 × 10^4^ cells per well), and infected with lentivirus, according to the manufacturer's protocol. Control (Ctrl) THP-1 cells and CRBN-knockdown (CRBN^KD^) THP-1 cells were selected in puromycin-containing (4–8 μg/ml) medium, and cultured as previously described (7).

### Generation of CRBN-Knockout Cell Line With CRISPR/Cas9

Guide RNA sequences for CRISPR/Cas9 were designed at the CRISPR design web site (http://crispr.mit.edu/), provided by the Feng Zhang Lab. Insert oligonucleotides for human CRBN gRNA is 5′-CACCGATATGCCTATCGAGAAGAAC-3′/5′-AAACGTTCTTCTCGATAGGCATATC-3′. The CRBN guide RNA targets the exon 2 of CRBN gene. The complementary oligonucleotides for guide RNAs (gRNAs) were annealed, and cloned into lenti CRISPR v2 vector (Addgene plasmid, Ca#52961). H1299 and MCF7 cells were transfected with lenti CRISPR v2/gRNA using Lipofectamine 2000, according to the manufacturer's instructions. Two days after transfection, cells were treated with 2 μg/ml of puromycin for 3 days. After 2 weeks, colonies were isolated with the 96-well plate, and the expression levels of CRBN were analyzed with western blot.

### Cellular Fractionation

Cellular fractionation assay was performed as previously described (19, 24). Briefly, THP-1 cells were treated with or without LPS (200 ng/ml) for different times. Cytosol and mitochondria fractions were isolated using a Cell Fractionation Kit (Ca# ab109719, Abcam), according to the manufacturer's protocol. Immunoblotting analysis was performed with anti-TRAF6, anti-CRBN, anti-IκB-α, and anti-GRIM19 antibodies.

### Immunofluorescence Microscopy

Confocal microscopy assay for subcellular localization of CRBN was performed as previously described (16, 22, 24). Briefly, THP-1 cells were treated with or without LPS (200 ng/ml) for 30 min, and stained with MitoTracker Red, anti-CRBN antibody, and DAPI, as described (16). Cells were imaged on a Zeiss LSM 710 laser-scanning confocal microscope (Carl Zeiss, Jena, Germany). For LC3 puncta assay, cells were cultured on glass coverslips for overnight. The cells were fixed with paraformaldehyde (4%), and treated with 0.2% Triton X-100 (0.2%) for cell permeabilization in ice for 30 min. Immunofluorescence microscopy assay was performed as previously described (22, 23). Slides were mounted in VECTASHIELD mounting medium (Vector Laboratories, Ca# H-1000), and examined under an LSM 710 laser-scanning confocal microscope (Carl Zeiss).

### Western Blotting and Immunoprecipitation (IP) Assays

Western blotting and immunoprecipitation (IP) assays were performed as previously described (7, 23–26). Briefly, expression vectors were transfected into HEK293T cells by using Lipofectamine 2000. Cells were harvested at 38 h after transfection and IP assay was performed with either anti-Flag or anti-Myc antibody. IP samples were separated by SDS-PAGE (6–10%), and immunoblotted with anti-HA, anti-Myc, or anti-Flag antibody. Ctrl and CRBN^KD^ THP-1 cells were treated with or without LPS (200 ng/ml) for different times. Immunoblotting assay was performed with antibodies, anti-LC3A/B, and anti-GAPDH. Ctrl H1299, CRBN^KO^ H1299, Ctrl MCF7, and CRBN^KO^ MCF7 cells were treated with or without 3-MA (5 mM) and CQ (10 μM), in the presence or absence of LPS (5 μg/ml), for 6 h. Immunoblotting assay was performed with antibodies, anti-LC3A/B, anti-CRBN, and anti-GAPDH.

### mROS Measurement

mROS measurement was performed as previously described (26), Briefly, cells were treated with or without LPS (200 ng/ml) for different times, washed with PBS, and incubated in RPMI medium with 2.5 mM MitoSOX Red for 15–30 min at 37°C. Cells were washed with warmed PBS and resuspended in cold PBS containing 1% FBS. To measure mitochondrial superoxide, flow cytometric analysis was performed with a FACScalibur apparatus (BD Biosciences, San Diego, CA, USA). All mROS experiments shown are representative of three independent experiments. For immunofluorescence microscopy, cells were mounted and examined under a LSM 710 laser-scanning confocal microscope (Carl Zeiss).

### Salmonella Infection Assay

Salmonella infection was performed as previously described (23, 25, 27). Briefly, cells were cultured with antibiotics-free RPMI 1640 medium in the presence of 20 ng/ml phorbol 12-myristate13-acetate (PMA) for 24 h. Cells (7 × 10^5^ cells/ml) were seeded into culture wells. The next day, non-adherent cells were removed, and antibiotics-free medium was freshly replaced and infected with Salmonella typhimurium wild type (14028s strain) at a multiplicity of infection of 10 bacteria/cell. Plates were centrifuged at 200 × g for 5 min and incubated at 37°C for 30 min. The medium was exchanged with fresh medium supplied with 20 μg/ml gentamicin, and further incubated for different times. Cells were harvested and lysed with DPBS containing 0.5% deoxycholate.

### Wound-Healing and Transwell Migration Assay

A wound-healing assay was performed as previously described (22, 23). Briefly, cells were grown to confluence in 12-well plates, scratched to form a wide gap (~400 μm), and washed with culture medium. Cells were treated with vehicle (DMSO, <0.2%), 5 mM 3-MA, or 10 μM CQ in the presence or absence of 5 μg/ml LPS. Cell images were captured at different times. In order to analysis cell migration, 5 × 10^4^ cells per well were prepared and re suspended in DMEM containing vehicle, 5 mM 3-MA, or 10 μM CQ in the presence or absence of 5 μg/ml LPS. Cells were seeded into the top chambers of transwells inserts (8 μm pore; Corning, 3422), and 10% FBS DMEM medium were added to the bottom chambers. After incubating the cells overnight, non-migrated cells from the top champer were removed, and migrated cells existed in the bottom chamber were fixed. To visualize the nuclei, fixed cells were stained with crystal violet. Experiments were performed in triplicate, and repeated twice.

### Statistical Analysis

Data are represented as mean ± SEM of the mean from triplicate samples. Statistical analysis was performed by ANOVA or Student's *t*-test using GraphPad Prism 5.0 (GraphPad Software, San Diego, CA, USA).

## Results

### CRBN Negatively Regulates mROS Production and Bactericidal Activity

**Figure 1:**
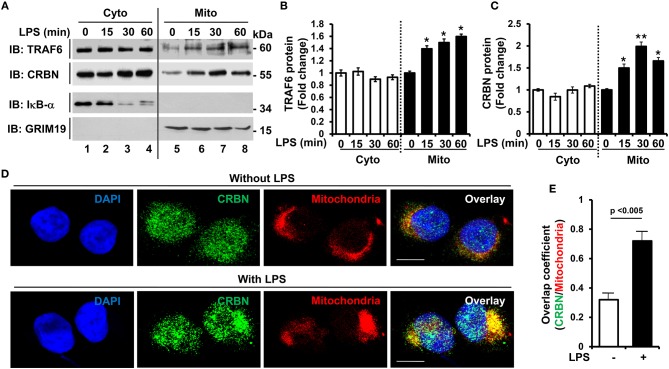
CRBN is localized in mitochondria in response to TLR4 stimulation. **(A)** THP-1 cells were treated with or without LPS (200 ng/ml) for different times, as indicated. Subcellular fractions, cytosolic (Cyto) and mitochondrial (Mito) fractions, were isolated, and western blot analysis was performed with antibodies, anti-TRAF6, anti-CRBN, anti-IκB-α as a cytosolic marker, and anti-GRIM19 as a mitochondrial marker. **(B,C)** Band intensity of TRAF6 **(B)**, and CRBN **(C)** was analyzed with Image J program. Data shown are averages from a minimum of 3 independent experiments (± SEM). *p < 0.05, **p < 0.01, when compared to that of without LPS. **(D)** THP-1 cells were treated with or without LPS (200 ng/ml) for 30 min, and confocal microscopy analysis was performed as described in the Materials and Methods (scale bar = 20 μm). **(E)** Overlap coefficients of CRBN and mitochondria were calculated, and represented [*n* = (10–15) cells].

It has been reported that TRAF6 is implicated in the production of mROS through the recruitment of TRAF6 to mitochondria upon TLR1/2/4 stimulation, and involved in bactericidal activity (19). Recently, we reported that CRBN interacts with TRAF6, and is negatively involved in TLR4 signaling by the attenuation of TRAF6 ubiquitination (7). Based on these results, we raised the possibility that CRBN is implicated in TLR4-induced mROS production, and thereby involved in bactericidal activity. THP-1 cells were stimulated with LPS for different times, and subcellular localization of CRBN was examined by western blotting. Consistent with previous reports (19), the localization of TRAF6 into mitochondrial fractions was significantly modulated by LPS stimulation (Figures 1A,B: TRAF6 and Figure 1B, closed bars). We found that CRBN was dominantly existed in the cytosolic fraction, but also marginally in the mitochondrial fraction in the absence of LPS stimulation (Figures 1A,B: CRBN in lane 1 and 5). This was consistent with a previous report (16). Interestingly, upon LPS stimulation, the localization of CRBN into mitochondria was significantly increased (Figures 1A,B: CRBN in lanes 6–8, and Figure 1C, closed bars). To verify the results, cellular localization of CRBN was measured by immunofluorescence microscopy. Consistently, the mitochondrial localization of CRBN was markedly enhanced in THP-1 cells treated with LPS (Figure 1D, without LPS vs. with LPS). The co-localization between CRBN and mitochondria could be verified by calculating overlap coefficient (Figure 1E, open bar vs. closed bar), indicating that the mitochondrial localization of CRBN is increased in response to TLR4 stimulation.

**Figure 2:**
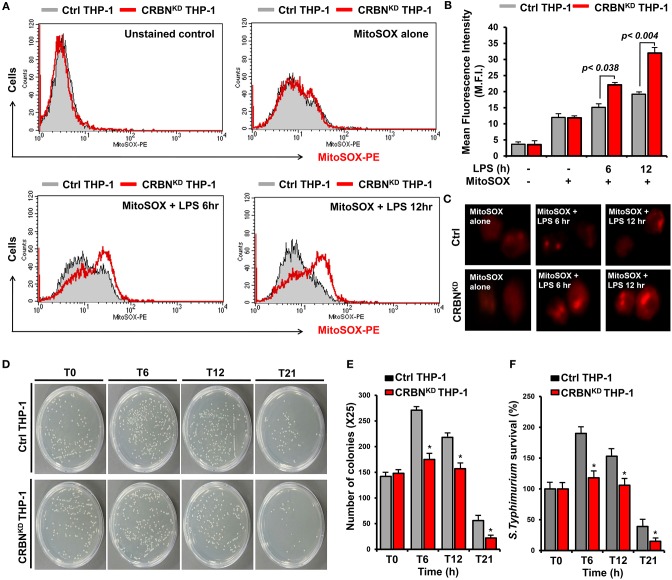
CRBN-knockdown THP-1 cells exhibit increases of mROS levels and bactericidal activity. **(A,B)** Control (Ctrl) and CRBN-knockdown (CRBN^KD^) THP-1 cells were treated with or without LPS for different times of 6 and 12 h, stained with MitoSOX-PE, and analyzed by flow cytometry **(A)**. Data are presented as the mean fluorescence intensity (M.F.I) ± SEM from triplicate samples **(B)**. **(C)** Ctrl and CRBN^KD^ THP-1 cells were treated with or without LPS for different times, stained with MitoSOX-PE, and analyzed by immunofluorescence microscopy. Data are representative of three independent replicates. **(D–F)** Ctrl and CRBN^KD^ THP-1 cells were infected with Salmonella wild type (14028s strain) at a multiplicity of infection of 10 bacteria/cell, as described in the Methods. Cells were lysed with 0.5% deoxycholate in Dulbecco's PBS. Bacteria were diluted (×25), and plated onto LB agar **(D)**. The number of colonies was counted and presented **(E)**. Percentage survival was obtained by dividing the number of bacteria recovered after 0 h (T0), 6 h (T6), 12 h (T12), or 21 h (T21) by the number of bacteria present at time 0 h (T0) and multiplying by 100. All error bars represent mean ±SEM of 3 independent experiments **(F)** *p < 0.05.

We next examined whether CRBN is implicated with mROS production induced by TLR4 stimulation, and thereby functionally involved in bactericidal activity. In order to do this, we generated CRBN-knockdown (CRBN^KD^) THP-1 cells by using lentiviral particles containing CRBN-shRNA, as well as control (Ctrl) THP-1 cells by using control lentiviral particles (Figure S1), as described in the Supplementary Information (SI). Cells were treated for different times with or without LPS, and then mROS were measured by flow cytometic analysis. The mROS levels in CRBN^KD^ THP-1 cells treated with LPS were significantly elevated, as compared with those of Ctrl THP-1 cells treated with LPS (Figures 2A,B, CRBN^KD^ THP-1 treated with LPS vs. Ctrl THP-1 treated with LPS). Moreover, the results were also confirmed by immunofluorescence microscopy (Figure 2C, CRBN^KD^ THP-1 treated with LPS vs. Ctrl THP-1 treated with LPS), supposing that CRBN may be negatively involved in the production of mROS induced by TLR4 stimulation.

The mROS generation regulated by TRAF6-ECSIT complex critically contributes to macrophage bactericidal activity (19). Consistently, we also found that ECSIT^KD^ or TRAF6^KD^ THP-1 cells exhibit marked decrease of mROS levels (Figures S2A–C), as compared with those of Ctrl THP-1 cells (Figures S2A–C, Ctrl THP-1 vs. ECSIT^KD^ or TRAF6^KD^ THP-1 cells). Furthermore, the survival of S. typhimurium was significantly increased in ECSIT^KD^ or TRAF6^KD^ THP-1 cells (Figure S3), strongly supporting that ECSIT and TRAF6 proteins might be essential for bactericidal activity mediated by mROS in response to TLRs stimulation. Based on these results, we further examined whether the increase of mROS in CRBN^KD^ THP-1 cells affects bactericidal activity. Ctrl and CRBN^KD^ THP-1 cells were infected with 10 MOI of S. typhimurium, and the survival of the bacterium was measured. The number of colonies was significantly decreased in CRBN^KD^ THP-1 cells, as compared with those of Ctrl THP-1 cells (Figures 2D,E, Ctrl THP-1 vs. CRBN^KD^ THP-1 cells). Moreover, the survival of S. typhimurium was significantly attenuated in CRBN^KD^ THP-1 cells (Figure 2F, Ctrl THP-1 vs. CRBN^KD^ THP-1 cells). These results suggest that CRBN might be negatively implicated in mROS production, and thereby involved in bactericidal activity, although a more detailed molecular mechanism is further required.

### CRBN Interacts With TRAF6 and ECSIT, and Inhibits the Ubiquitination of ECSIT

**Figure 3:**
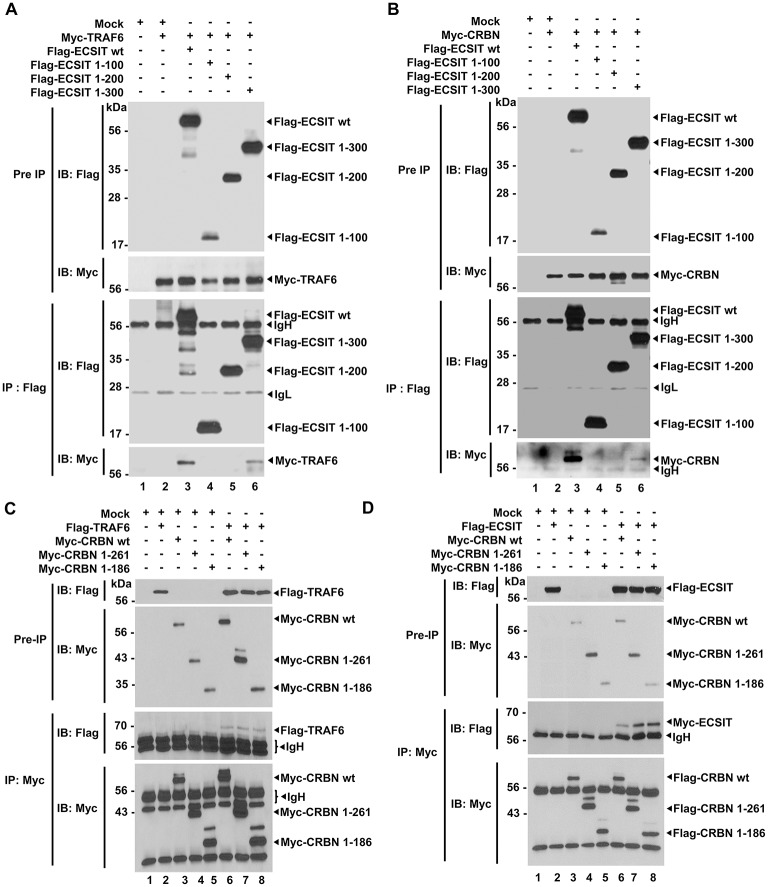
CRBN interacts with ECSIT and TRAF6. **(A)** HEK293T cells were transfected with vector control (Mock), Myc-TRAF6, Flag-ECSIT wild type (wt), Flag-ECSIT 1-100, Flag-ECSIT1-200, or Flag-ECSIT 1-300, as indicated. At 38 h after transfection, transfected cells were extracted, immunoprecipitated with anti-Flag antibody, and then an IB assay was performed with anti-Flag or anti-Myc antibody. **(B)** HEK293T cells were transfected with Mock, Myc-CRBN, Flag-ECSIT wt, Flag-ECSIT 1-100, Flag-ECSIT 1-200, or Flag-ECSIT 1-300, as indicated. At 38 h after transfection, transfected cells were extracted, immunoprecipitated with anti-Flag antibody, and then an IB assay was performed with anti-Flag or anti-Myc antibody. **(C)** HEK293T cells were transfected with Mock, Flag-TRAF6, Myc-CRBN wt, Myc-CRBN 1-261, or Myc-CRBN 1-186, as indicated. At 38 h after transfection, transfected cells were extracted, immunoprecipitated with anti-Myc antibody, and then an IB assay was performed with anti-Flag or anti-Myc antibody. **(D)** HEK293T cells were transfected with Mock, Flag-ECSIT, Myc-CRBN wt, Myc-CRBN 1-261, or Myc-CRBN 1-186, as indicated. At 38 h after transfection, transfected cells were extracted, immunoprecipitated with anti-Myc antibody, and then an IB assay was performed with anti-Flag or anti-Myc antibody.

Having shown the above results, we next explored the molecular mechanism by which CRBN is negatively involved in mROS generation induced by TLR4 stimulation. TRAF6 is functionally associated with mROS generation through the ubiquitination of mitochondrial ECSIT protein (19). Moreover, we recently reported that CRBN interacts with TRAF6, and induces the attenuation of ubiquitination of TRAF6 (7). Therefore, we hypothesized that the interaction of CRBN with TRAF6 might affect the ubiquitination of ECSIT. To examine this possibility, we first determined the molecular association of TRAF6, ECSIT, and CRBN proteins. We transiently transfected Flag-ECSIT wild type (wt) and Flag-ECSIT truncated mutants (Figure S5A), along with Myc-TRAF6 (Figure 3A) or Myc-CRBN (Figure 3B) into HEK293T cells, and immunoprecipitation (IP) assay was performed with anti-Flag antibody. Interestingly, Myc-TRAF6 or Myc-CRBN was co-precipitated with Flag-ECSIT wt and Flag-ECSIT 1–300 (Figure 3A, lanes 3 and 6; Figure 3B, lanes 3 and 6), but not with Flag-ECSIT 1-100 and Flag-ECSIT 1-200 (Figure 3A, lanes 4 and 5; Figure 3B, lanes 4 and 5), indicating that ECSIT 200-300 region is critical for the interaction with TRAF6 or CRBN, as depicted in Figure S5B. We next transiently transfected with Myc-CRBN wt and Myc-CRBN truncated mutants (Figure S5C), along with Flag-TRAF6 (Figure 3C) or Flag-ECSIT (Figure 3D) into HEK293T cells, and performed IP assay with anti-Myc antibody. Interestingly, Flag-TRAF6 and Flag-ECSIT were co-precipitated with Myc-CRBN wt, Myc-CRBN 1-261, and Myc-CRBN 1-186 (Figures 3C,D, lanes 6-8). These results suggest that CRBN interacts with TRAF6 and ECSIT through its N-terminal region, as depicted in Figure S5D.

**Figure 4:**
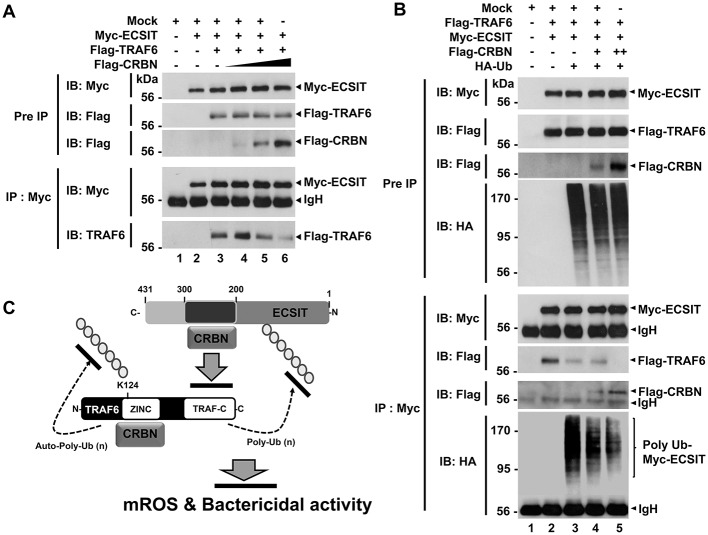
CRBN inhibits the ubiquitination of ECSIT. **(A)** HEK293T cells were transfected with Mock, Myc-ECSIT, Flag-TRAF6, or different concentrations of Flag-CRBN, as indicated. At 38 h after transfection, transfected cells were extracted, immunoprecipitated with anti-Myc antibody, and then an IB assay was performed with anti-Flag, anti-Myc, or anti-TRAF6 antibody. **(B)** HEK293T cells were transfected with Mock, Flag-TRAF6, Myc-ECSIT, HA-Ub, or different concentrations of Flag-CRBN, as indicated. At 38 h after transfection, transfected cells were extracted, immunoprecipitated with anti-Myc antibody, and then an IB assay was performed with anti-Flag, anti-Myc, or anti-HA antibody. **(C)** Model of how CRBN inhibits the production of mROS through the inhibition of the ubiquitination of ECSIT.

Having shown that TRAF6 and CRBN interacted with the ECSIT 200-300 region (Figures 3A,B), and TRAF6 and ECSIT interacted with the N-terminal region of CRBN (Figures 3C,D), we asked whether CRBN could interrupt the interaction between TRAF6 and ECSIT. Flag-TRAF6 and Myc-ECSIT vectors were transiently transfected into HEK293T cells, along with different concentrations of Flag-CRBN vector, as indicated in Figure 4A, and then IP assay was performed with anti-Myc antibody. As expected, Myc-ECSIT was strongly precipitated with Flag-TRAF6 in the absence of Flag-CRBN (Figure 4A, lane 3), whereas the interaction was gradually attenuated in the presence of Flag-CRBN (Figure 4A, lane 3 vs. lanes 4–6), indicating that CRBN interrupts the association of TRAF6 and ECSIT proteins. Based on the result, we further assessed whether CRBN could affect the ubiquitination of ECSIT. Flag-TRAF6, Myc-ECSIT, and HA-Ub vectors were transiently transfected into HEK293T cells along with different concentrations of Flag-CRBN vector, and then the ubiquitination of ECSIT was evaluated. Consistently, the ubiquitination of ECSIT was induced in the absence of Flag-CRBN (Figure 4B, lane 3), whereas attenuations of ECSIT ubiquitination were observed in the presence of CRBN (Figure 4B, lane 3 vs. lanes 4 and 5). Upon LPS stimulation, moreover, ubiquitination of endogenous ECSIT proteins was markedly enhanced in CRBN^KD^ THP-1 cells as compared to that of Ctrl THP-1 cells (Figure S4, lane 2 vs. lane 4), supporting the inhibitory role of CRBN in ECSIT ubiquitination induced by TLR4 stimulation. Together, these results suggest that CRBN interacts with ECSIT and TRAF6, as depicted in Figure 4C. As we previously reported (7), the interaction of CRBN with TRAF6 induces the inhibition of auto-ubiquitination of TRAF6, and that leads to the inhibition of TLR4-induced activation of NF-κB. In addition, we demonstrate in this study that the interaction of CRBN with ECSIT interrupts the association of ECSIT-TRAF6 complex, and inhibits the ubiquitination of ECSIT, and thereby negatively regulates mROS production and bactericidal activity.

### CRBN Inhibits Autophagy Activation Through the Inhibition of BECN1 Ubiquitination

**Figure 5:**
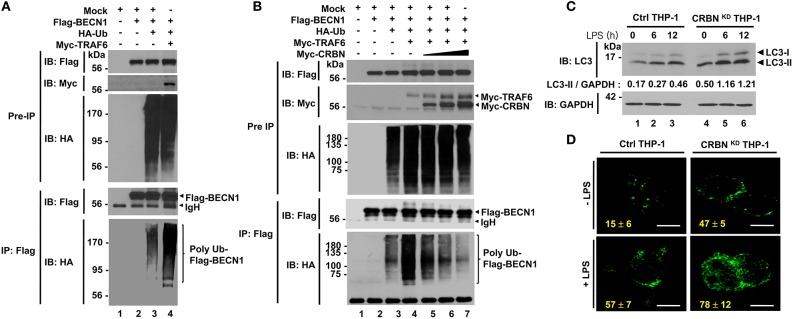
CRBN inhibits the ubiquitination of BECN1. **(A)** HEK293T cells were transfected with Mock, Flag-BECN1, HA-Ub, or Myc-TRAF6, as indicated. At 38 h after transfection, transfected cells were extracted, immunoprecipitated with anti-Flag antibody, and then an IB assay was performed with anti-Flag, anti-Myc, or anti-HA antibody. **(B)** HEK293T cells were transfected with Mock, Flag-BECN1, HA-Ub, Myc-TRAF6, or different concentrations of Myc-CRBN, as indicated. At 38 h after transfection, transfected cells were extracted, immunoprecipitated with anti-Flag antibody, and then an IB assay was performed with anti-Flag, anti-Myc, or anti-HA antibody. **(C)** Control (Ctrl) and CRBN^KD^ THP-1 cells were treated with or without LPS (200 ng/ml) for different times, as indicated. Cells were lysed and subjected to SDS-PAGE, followed by immunoblotting with LC3-I/-II or GAPDH antibody. Band intensity was quantified using Image J software. Quantitative data were calculated from 3 independent experiments. Data are shown as mean ± SEM. **(D)** Ctrl and CRBN^KD^ THP-1 cells were treated with or without LPS (200 ng/ml) for 6 h, and then fixed. Immunofluorescence assay was performed with anti-LC3 antibody. Digital images were captured with confocal microscopy, and the number of LC3-puncta was scored. Quantification represents the mean ± SEM of puncta per cell (*n* = 5) from 3 independent experiments. Scale bar: 10 μm.

TRAF6 coordinates the activation of autophagy through the ubiquitination of BECN1 (21–23, 28). Having shown that CRBN interacts with TRAF6 and inhibits the ubiquitination of ECSIT, we raised the possibility that CRBN is also implicated in autophagy activation through the regulation of TRAF6-mediated ubiquitination of BECN1. Consistent with previous results (21–23), we found that BECN1 interacted with TRAF6 (Figure S6), and ubiquitination of BECN1 was significantly enhanced in the presence of TRAF6 (Figure 5A, lane 3 vs. lane 4). Interestingly, the ubiquitination of BECN1 was gradually attenuated in the presence of CRBN, as compared with that in the absence of CRBN (Figure 5B, lane 4 vs. lanes 5–7), indicating that CRBN inhibits the TRAF6-mediated ubiquitination of BECN1. To examine whether CRBN is functionally involved in the autophagy activation induced by TLR4, Ctrl and CRBN^KD^ THP-1 cells were treated with LPS for different time periods, and the ratio between the amounts of LC3-I and LC3-II was then monitored by western blotting. Levels of LC3-II were significantly elevated in CRBN^KD^ THP-1 cells treated with LPS, as compared to those in Ctrl THP-1 cells treated with LPS (Figure 5C). Consistently, the number of autophagy in CRBN^KD^ THP-1 cells was significantly higher than that in Ctrl THP-1 cells (Figure 5D, Ctrl THP-1 vs. CRBN^KD^ THP-1).

**Figure 6:**
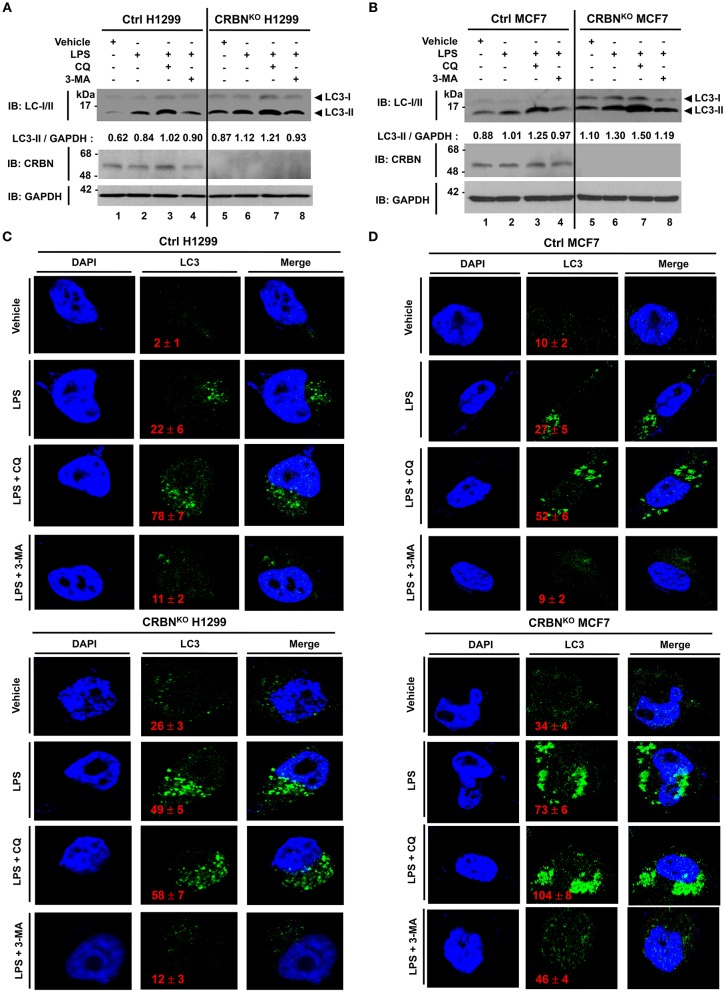
CRBN-knockout H1299 and MCF7 cancer cells exhibit the increase of autophagy activation. **(A)** Control (Ctrl) and CRBN-knockout (CRBN^KO^) H1299 cells were treated with or without LPS (5 μg/ml) in the presence or absence of CQ (10 μM) and 3-MA (5 mM) for 6 h, as indicated. Cells were lysed and subjected to SDS-PAGE, followed by immunoblotting with LC3-I/-II, CRBN, and GAPDH antibody. Band intensity was quantified using Image J software. Quantitative data were calculated from 3 independent experiments. Data are shown as mean ± SEM. **(B)** Ctrl and CRBN^KO^ MCF7 cells were treated with or without LPS (5 μg/ml) in the presence or absence of CQ (10 μM) and 3-MA (5 mM) for 6 h, as indicated. Cells were lysed and subjected to SDS-PAGE, followed by immunoblotting with LC3-I/-II, CRBN, and GAPDH antibody. Band intensity was quantified using Image J software. **(C)** Ctrl and CRBN^KO^ H1299 cells were treated with or without LPS (5 μg/ml) in the presence or absence of CQ (10 μM) and 3-MA (5 mM) for 6 h, as indicated, and then fixed. Immunofluorescence assay was performed with anti-LC3 antibody. Digital images were captured with confocal microscopy, and the number of LC3-puncta was scored. Quantification represents the mean ± SEM of puncta per cell (*n* = 5) from 3 independent experiments. **(D)** Ctrl and CRBN^KO^ MCF7 cells were treated with or without LPS (5 μg/ml) in the presence or absence of CQ (10 μM) and 3-MA (5 mM) for 6 h as indicated, and then fixed. Immunofluorescence assay was performed with anti-LC3 antibody. Digital images were captured with confocal microscopy, and the number of LC3-puncta was scored. Quantification represents the mean ± SEM of puncta per cell (*n* = 5) from 3 independent experiments.

To verify the functional role of CRBN in autophagy activation, we generated CRBN-knockout (CRBN^KO^) H1299 human non-small cell lung carcinoma cells and CRBN^KO^ MCF7 breast cancer cells by using CRISPR-Cas9 gene-editing technology (Figure 6A, CRBN^KO^ H1299; Figure 6B, CRBN^KO^ MCF7), as described in the Materials and Methods. Cells were treated with or without LPS in the presence or absence of 3-methyladenine (3-MA), an inhibitor of phosphatidylinositol 3-kinases, and chloroquine (CQ), an inhibitor of autophagosomal degradation, for 6 h, and then the ratio between the amounts of LC3-I/LC3-II and GAPDH was monitored by western blotting. Upon TLR4 stimulation, levels of LC3-II were markedly elevated in CRBN^KO^ H1299 and CRBN^KO^ MCF7 cells, as compared to those in Ctrl H1299 and Ctrl MCF7 cells, respectively (Figures 6A,B, lane 2 vs. lane 6). As expected, significant accumulation of LC3-II protein levels could be detected in the treatment of the CQ (Figures 6A,B, lanes 3 and 7), whereas marked attenuations could be seen in the treatment of 3-MA (Figures 6A,B, lanes 4 and 8). Consistently, immunofluorescence microscopy results revealed that the number of autophagosomes in CRBN^KO^ H1299 and CRBN^KO^ MCF7 cells treated with LPS was significantly higher than those in Ctrl H1299 and Ctrl MCF7 cells, respectively (Figure 6C, Ctrl H1299 vs. CRBN^KO^ H1299 treated with LPS; Figure 6D, Ctrl MCF7 vs. CRBN^KO^ MCF7 treated with LPS). Moreover, autophagosomes were significantly elevated in both cells treated with CQ (Figure 6C, LPS vs. LPS plus CQ in Ctrl H1299 and CRBN^KO^ H1299; Figure 6D, LPS vs. LPS plus CQ in Ctrl MCF7 and CRBN^KO^ MCF7), whereas marked attenuations could be seen in the treatment of 3-MA (Figure 6C, LPS vs. LPS plus 3-MA in Ctrl H1299 and CRBN^KO^ H1299; Figure 6D, LPS vs. LPS plus 3-MA in Ctrl MCF7 and CRBN^KO^ MCF7). These results strongly suggest that CRBN is a negative regulator of autophagy activation.

### CRBN-Knockout Cancer Cells Exhibit Increases of the Ability of Migration and Invasion

**Figure 7:**
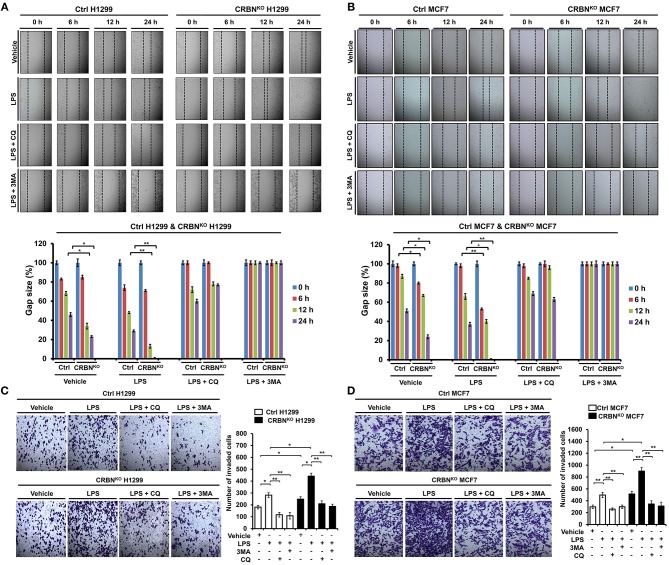
CRBN-knockout H1299 and MCF7 cancer cells exhibit the enhancement of cell migration and invasion. **(A)** Ctrl and CRBN^KO^ H1299 cells were seeded into 12-well cell culture plates, cultured in DMEM supplemented with 10% FBS, and allowed to grow to near confluence. Confluent monolayers were carefully wounded with a sterile yellow Gilson-pipette tip, cellular debris was gently washed away with culture medium, and the wound was then treated with vehicle (DMSO, <0.2% in culture medium), LPS (5 μg/ml), 3-MA (5 mM) plus LPS (5 μg/ml), and CQ (10 μM) plus LPS (5 μg/ml) for different time periods, as indicated. One representative experiment was shown. The residual gap between the migrating cells from the opposing wound edge was expressed as a percentage of the initial scraped area (± SEM, *n* = 3) *p < 0.05, **p < 0.01. **(B)** Ctrl and CRBN^KO^ MCF7 cells were seeded into 12-well cell culture plates, cultured in DMEM supplemented with 10% FBS, and allowed to grow to near confluence. Confluent monolayers were carefully wounded with a sterile yellow Gilson-pipette tip, cellular debris was gently washed away with culture medium, and the wound was then treated with vehicle (DMSO <0.2% in culture medium), LPS (5 μg/ml), 3-MA (5 mM) plus LPS (5 μg/ml), and CQ (10 μM) plus LPS (5 μg/ml) for different time periods, as indicated. One representative experiment was shown. The residual gap between the migrating cells from the opposing wound edge was expressed as a percentage of the initial scraped area (± SEM, *n* = 3). *p < 0.05, **p < 0.01. **(C)** Ctrl and CRBN^KO^ H1299 cells were suspended in DMEM containing vehicle, LPS (5 μg/ml), 3-MA (5 mM) plus LPS (5 μg/ml), and CQ (10 μM) plus LPS (5 μg/ml), and added to the top chambers of the transwells in 24-well plates. After an overnight incubation, cells were fixed and stained with crystal violet. Number of cell migration was counted, and results are presented as mean ± SEM of 3 independent experiments *p < 0.05, **p < 0.01. **(D)** Ctrl and CRBN^KO^ MCF7 cells were suspended in DMEM containing vehicle, LPS (5 μg/ml), 3-MA (5 mM) plus LPS (5 μg/ml), and CQ (10 μM) plus LPS (5 μg/ml), and added to the top chambers of the transwells in 24-well plates. After an overnight incubation, cells were fixed, and stained with crystal violet. Number of cell migration was counted, and results are presented as mean ± SEM of 3 independent experiments *p < 0.05, **p < 0.01.

It has been reported that autophagy promotes TLR4- and TLR3-induced migration and invasion of lung cancer cells (22). Having shown that CRBN^KO^ H1299 and CRBN^KO^ MCF7 cells exhibited increases of autophagy activation induced by TLR4 stimulation (Figure 6), we further examined whether CRBN affected the migration and invasion of cancer cells. In order to do this, wound healing and transwell invasion assays were performed to measure the capacity of cancer migration and invasion. The migration of CRBN^KO^ H1299 and CRBN^KO^ MCF7 cells treated with vehicle was increased in a time-dependent manner, as compared with those of Ctrl cancer cells (Figure 7A, vehicle: Ctrl H1299 vs. CRBN^KO^ H1299; Figure 7B, vehicle: Ctrl MCF7 vs. CRBN^KO^ MCF7). Moreover, similar results could be seen in CRBN^KO^ H1299 and CRBN^KO^ MCF7 cells treated with LPS (Figure 7A, LPS: Ctrl H1299 vs. CRBN^KO^ H1299; Figure 7B, LPS: Ctrl MCF7 vs. CRBN^KO^ MCF7). As expected, the treatment of 3-MA or CQ in the presence of LPS induced the marked inhibition of migration in cancer cells (Figures 7A,B, LPS vs. LPS plus 3-MA or LPS plus CQ). Furthermore, transwell invasion assay revealed significant increases of cellular invasive ability in CRBN^KO^ H1299 and CRBN^KO^ MCF7 cells treated with vehicle or LPS, as compared to those of Ctrl cancer cells (Figure 7C, Ctrl H1299 vs. CRBN^KO^ H1299 in Vehicle or LPS; Figure 7D, Ctrl MCF7 vs. MCF7^KO^ H1299 in Vehicle or LPS), whereas the marked attenuation could be detected in the treatment of CQ or 3-MA (Figures 7C,D, LPS vs. LPS plus CQ or LPS plus 3-MA). Consistent with the previous report (22), TLR4 stimulation induces cancer migration and invasion through the autophagy activation. Meanwhile, our results demonstrate that CRBN negatively regulates cancer migration and invasion through the inhibition of autophagy activation induced by TLR4 stimulation.

## Discussion

The phagocytic response is a necessary effector function for the destruction of intracellular microbes in innate immune system through the production of ROS (19, 29, 30). Along with the phagosomal NADPH-oxidase-dependent respiratory burst to produce ROS, the mitochondrial oxidative phosphorylation machinery critically contributes to the generation of ROS (17, 29, 30). Although the molecular mechanism by which mROS is produced and regulated in mitochondria remains to be clearly understood, recent evidence has suggested that TLR signals function to the mROS production through the translocation of mitochondria to phagosomes, which is regulated by the TRAF6-ECSIT complex (19), indicating the association of TRAF6-ECSIT proteins might play a pivotal role for mROS generation, and is functionally associated with bactericidal activity in innate immune cells. Consistently, we found that ECSIT^KD^ or TRAF6^KD^ THP-1 cells exhibit marked decrease of mROS levels (Figure S2) and lead to the suppression of bactericidal activity, as compared with those of Ctrl THP-1 cells (Figure S3), indicating that ECSIT and TRAF6 proteins are essential for bactericidal activity mediated by mROS in response to TLRs stimulation.

**Figure 8:**
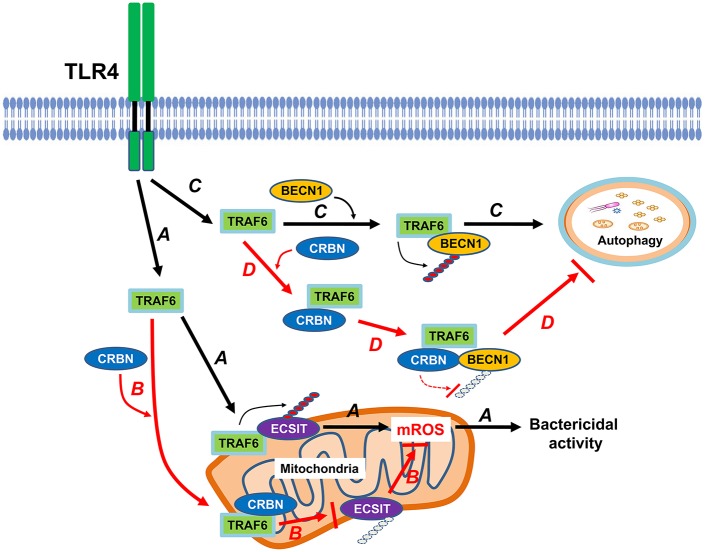
CRBN is negatively involved in mROS generation and autophagy activation in response to TLR4 stimulation. Engagement of TLR4 ligand leads to concomitant trafficking of TRAF6 to mitochondria, where TRAF6 interacts with and ubiquitinates ECSIT. ECSIT ubiquitination alters OXPHOS activity, induces the generation of mROS, and that is implicated in bactericidal activity (indicated as arrow **A**). However, if CRBN interacts with TRAF6, the interaction interrupts the association of TRAF6-ECSIT, and inhibits the ubiquitination of ECSIT (indicated as arrow **B**). Upon TLR4 stimulation, TRAF6 interacts with BECN1, and induces ubiquitination of BECN1. Ubiquitinated BECN1 is implicated in the activation of autophagy (indicated as arrow **C**). In contrast, the interaction between CRBN and TRAF6 inhibits TRAF6 E3 ligase activity and that leads to the inhibition of BECN1 ubiquitination, and results in the suppression of autophagy activation (indicated as arrow **D**).

CRBN is multifunctional and localized in subcellular compartments, including the nucleus, cytoplasm, and endoplasmic reticulum (ER) (3–5). It has been reported that CRBN is localized in mitochondria, and functions as protection against oxidative stress (16). Recently, we reported that CRBN negatively regulates TLR4 signaling through the attenuation of the ubiquitination of TRAF6 (7). Based on these findings, we investigated the functional role of CRBN in mitochondria for mROS production induced by TLR4 stimulation. We found that CRBN interacts with ECSIT and TRAF6 proteins, and the CRBN-ECSIT interaction interrupts the association of TRAF6 with ECSIT, thereby leading to the inhibition of the ubiquitination of ECSIT. Interestingly, we also found that the mROS level was significantly higher in CRBN^KD^ THP-1 cells than in Ctrl THP-1 cells, suggesting that CRBN might be negatively involved in the production of mROS through the inhibition of ECSIT ubiquitination. In terms of functional aspect related to mROS, CRBN^KD^ THP-1 cells exhibited resistance against Salmonella infection, strongly indicating the negative regulation of CRBN on bactericidal activity induced by TLR4 stimulation. These results suggest that CRBN negatively regulates mROS generation induced by TLR4 stimulation, and thereby involves bactericidal activity. In summary, as Figure 8A shows, engagement of TLR4 ligand leads to concomitant trafficking of TRAF6 to mitochondria, where TRAF6 interacts with and ubiquitinates ECSIT. ECSIT ubiquitination alters OXPHOS activity, induces the generation of mROS, and that is implicated in bactericidal activity (19). Our results demonstrate that CRBN interacts with TRAF6, interrupts the association of TRAF6-ECSIT, and inhibits the ubiquitination of ECSIT (Figure 8B). This inhibitory response might be critically affected by mROS generation to be related to ECSIT-TRAF6 complex in mitochondria, and thereby negatively implicated in bactericidal activity (Figure 8B).

Another issue to be explored in this study is whether CRBN is involved in autophagy activation induced by TLR4, and implicated in cancer progression. Autophagic flux is triggered and induced by various factors, such as hypoxia, nutrient deprivation, and infections (31, 32). Autophagy plays a complex and highly context-dependent role in tumorigenesis (33). In the metastatic cascade, multi-functions of autophagy have also been suggested (34). Furthermore, a recent report has shown that autophagy functions to TLR4- and TLR3-triggered progression of lung cancer cells through enhancing TRAF6 ubiquitination (22), indicating the pivotal role of autophagy in cancer progression. Since CRBN-TRAF6 interaction inhibited the TRAF6-mediated ubiquitination of ECSIT (Figure 4), we could assume that CRBN might affect the ubiquitination of BECN1 by TRAF6. As expected, CRBN negatively affected by the ubiquitination of BECN1, and CRBN^KD^ THP-1 cells exhibited the enhancement of autophagy activation in response to LPS stimulation. Moreover, CRBN^KO^ H1299 and CRBN^KO^ MCF7 cells revealed marked increases of autophagy activation in response to LPS stimulation. Although the direct evidence of CRBN being capable of affecting cancer progression is insufficient, many reports have suggested that CRBN is closely related to the proliferation and metabolism of normal and tumor cells (6, 35–37). To expand the multifunctional roles of CRBN in cancer progression induced by autophagy, we examined the abilities of cancer cell migration and invasion in CRBN^KO^ H1299 and CRBN^KO^ MCF7 cells. Interestingly, CRBN^KO^ H1299 and CRBN^KO^ MCF7 cells demonstrated the increase of cell migration capacity, as compared with those of control cancer cells. Moreover, similar results could be seen in cell invasion assay, indicating that CRBN is negatively implicated in cancer progression, presumably through the inhibition of autophagy activation. In summary, as depicted in Figure 8C, upon TLR4 stimulation, TRAF6 interacts with BECN1, induces the ubiqutination of BECN1, and that is involved in autophagy activation (21–23, 28). In current studies, we found that CRBN interacted with TRAF6. The interaction of CRBN with TRAF6 significantly led to attenuate the ubiquitination of BECN1 mediated by TRAF6, and thereby inhibited the activation of autophagy (Figure 8D).

So far, many studies on CRBN have focused on the multiple effects of immunomodulatory imide drugs (IMiDs) (6, 37–39). It has also been suggested that the cellular expression of CRBN influences cell metabolism and leads to disease in the absence of IMiDs, indicating the various functions and cellular mechanisms of CRBN (38). Nevertheless, the molecular mechanisms involved in these cellular processes are still poorly understood. We expect that our results will contribute to understanding the various cellular functions of CRBN and pathological mechanisms of inflammatory diseases induced by innate signals, as well as cancer diseases regulated by autophagy activation. Additionally, this understanding might be helpful for the development of therapeutic targets for combined clinical treatment targeted at inflammation and cancer.

## Author Contributions

EC and K-YL: conception and design of study. M-JK and YM: acquisition of data. M-JK, YM, J-HS, EC, and K-YL: analysis and interpretation of data. EC and K-YL: drafting the manuscript. M-JK, YM, J-HS, EC, and K-YL: important intellectual content. M-JK, YM, J-HS, EC, and K-YL: approval of manuscript.

### Conflict of Interest Statement

The authors declare that the research was conducted in the absence of any commercial or financial relationships that could be construed as a potential conflict of interest.
