# Supplementary material for: CRBN Is a Negative Regulator of Bactericidal Activity and Autophagy Activation Through Inhibiting the Ubiquitination of ECSIT and BECN1
Source: Front Immunol. 2019 Sep 18;10:2203. doi: 10.3389/fimmu.2019.02203 (PMC6759600; doi:10.3389/fimmu.2019.02203)
Supplement: Supplementary file 1 [file Data_Sheet_1.docx]

**Supplementary Information**

The supplementary Material includes Supplementary Materials and Methods, and Figures with legends

**Supplementary Materials and Methods**

**Generation of Knockdown THP-1 cells**

Lentivirus containing small hairpin RNA (shRNA) targeting human CRBN (sc-78528-V), human TRAF6 (sc-36717-V), human ECSIT (sc-77224-V), or control shRNA lentivirus (sc-108080) were purchased from Santa Cruz Biotechnology (Santa Cruz, CA, USA). THP-1 cells were cultured in wells of a 24-well plate (2 × 10^4^ cells per well), and infected with lentivirus, according to the manufacturer’s protocol. Ctrl THP-1 cells, CRBN-knockdown (CRBN^KD^) THP-1 cells, TRAF6-knockdown (TRAF6^KD^) THP-1 cells, and ECSIT-knockdown (ECSIT^KD^) THP-1 cells were selected in (4–8) μg/ml puromycin-containing medium, and cultured.

**mROS measurement**

Cells were treated with or without LPS (200 ng/ml) for different times. Culture medium was removed, and cells were washed with PBS, then incubated in serum-free RPMI 1640 medium for 15 to 30 min at 37 °C with MitoSOX Red (2.5 mM final concentration), to measure mitochondrial superoxide. Cells were washed with warmed PBS (37 °C), removed from plates by pipetting with cold PBS containing 1 mM EDTA, pelleted at 1,500 rpm for 3 min, immediately resuspended in cold PBS containing 1% FBS, and subjected to fluorescence-activated cell sorting analysis using a FACS calibur apparatus (BD Biosciences, San Diego, CA,USA). Data are presented as the mean fluorescence intensity (M.F.I) ± SEM from triplicate samples.

**Salmonella infection assay**

Cells were cultured in fresh RPMI 1640 complete medium without antibiotics in the presence of phorbol 12-myristate13-acetate (PMA, 20 ng/ml) for 24 h, and seeded into culture wells at a concentration of 7 × 10^5^ cells/ml. The next day, non-adherent cells were removed, and medium was replaced with fresh complete medium without antibiotics. Cells were infected with Salmonella typhimurium wild type (14028s strain; a kind gift from Dong woo Shin, Sungkyunkwan University, Korea) at a multiplicity of infection of 10 bacteria/cell. Culture plates were centrifuged at 200 × g for 5 min, and incubated at 37°C for 30min, to allow phagocytosis to occur. The medium was then replaced with fresh medium containing gentamicin (20 μg/ml), and incubated for different time periods. Total cell population in the well was harvested. An aliquot of the harvested cell population was centrifuged, and macrophages were lysed by 0.5% deoxycholate in Dulbecco’s phosphate-buffered saline (DPBS).

**Immunoprecipitation (IP) assays**

HEK293T cells were transfected with vector control (Mock), Myc-BECN1, or Flag-TRAF6, as indicated. At 38 h after transfection, transfected cells were extracted, immunoprecipitated with anti-Flag antibody, and then an IB assay was performed with anti-Flag or anti-Myc antibody.

**Endogenous IP and ubiquitination assay**

Ctrl or CRBN^KD^ THP-1 cells were stimulated with or without 200 μg/ml LPS for 60 min, and immunoprecipitation (IP) assay was performed with anti-ECSIT antibodies. Whole cell lysate and IP fractions were immuno-blotted with anti-Ub, anti-ECSIT, or anti-CRBN antibodies.

**Supplementary Figures**

**Supplementary Fig. S1: Generation of CRBN-knockdown THP-1 cells**

THP-1 cells were infected with a lentivirus containing shRNA targeted at human CRBN or control shRNA. Two weeks post-infection, endogenous expression of CRBN proteins were analyzed with anti-CRBN antibody in CRBN^KD^ and control (Ctrl) THP-1 cells.

**Supplementary Fig. S2: ECSIT-knockdown (ECSIT^KD^) THP-1 and TRAF6-knockdown (TRAF6^KD^) THP-1 cells exhibit decreases of mROS generation in response to LPS stimulation**

**(A and B)** THP-1 cells were infected with a lentivirus containing shRNA targeted at human ECSIT (**A**), human TRAF6 (**B**), or control (Ctrl) shRNA. Two weeks post-infection, endogenous expressions of ECSIT or TRAF6 proteins were analyzed with anti-ECSIT or anti-TRAF6 antibody in ECSIT^KD^, TRAF6^KD^, or Ctrl THP-1 cells. **(C**) Ctrl, ECSIT^KD^, or TRAF6^KD^ THP-1 cells were treated with or without LPS for different times, as indicated, stained with MitoSOX-PE, and analyzed by flow cytometry. Data are presented as the mean fluorescence intensity (M.F.I) ± SEM from triplicate samples. * p< 0.05, ** p < 0.01.

**Supplementary Fig. S3: ECSIT-knockdown (ECSIT^KD^) THP-1 and TRAF6-knockdown (TRAF6^KD^) THP-1 cells exhibit decreases of bactericidal activity**

Ctrl, ECSIT^KD^, and TRAF6^KD^ THP-1 cells were infected with Salmonella wild type (14028s strain) at a multiplicity of infection of 10 bacteria/cell, as described in the Methods. Cells were lysed with 0.5% deoxycholate in Dulbecco’s PBS. Bacteria were diluted (×50), and plated onto LB agar. The number of colonies was counted, and presented. Percentage survival was obtained by dividing the number of bacteria recovered after 0 h (T0), 6 h (T6), 12 h (T12), or 21 h (T21) by the number of bacteria present at time 0 h (T0, 101 ± 13) and multiplying by 100. All error bars represent mean ± SEM of 3 independent experiments.* p< 0.05, ** p < 0.01.

**Supplementary Fig. S4: Ubiquitination of endogenous ECSIT is enhanced in CRBN^KD^ THP-1 cells.**

Ctrl or CRBN^KD^ THP-1 cells were stimulated with or without 200 μg/ml LPS for 60 min, and immunoprecipitation (IP) assay was performed with anti-ECSIT antibody. Whole cell lysate and IP fractions were probed with the indicated antibodies.

**Supplementary Fig. S5: Schematic representation of the association of TRAF6, CRBN, and ECSIT proteins**

**(A**) Schematic of ECSIT wild type (WT) and ECSIT truncated mutants. **(B**) Schematic of the interaction domain of ECSIT with CRBN and TRAF6. (**C**) Schematic of CRBN wild type (WT) and CRBN truncated mutants. **(D**) Schematic of the interaction domain of CRBN with ECSIT and TRAF6.

**Supplementary Fig. S6: TRAF6 interacts with BECN1**

HEK293T cells were transfected with vector control (Mock), Myc-BECN1, or Flag-TRAF6, as indicated. At 38 h after transfection, transfected cells were extracted, immuneprecipitated with anti-Flag antibody, and then an IB assay was performed with anti-Flag or anti-Myc antibody.
