# Supplementary figures and images for: CRBN Is a Negative Regulator of Bactericidal Activity and Autophagy Activation Through Inhibiting the Ubiquitination of ECSIT and BECN1
Source: Front Immunol. 2019 Sep 18;10:2203. doi: 10.3389/fimmu.2019.02203 (PMC6759600; doi:10.3389/fimmu.2019.02203)

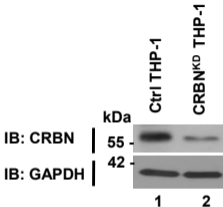

***Supplementary Figure S1***

Supplement: Supplementary file 2 [file Image_1.pdf]

**A**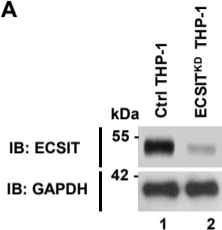**B**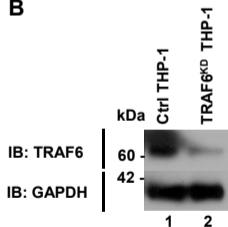**C**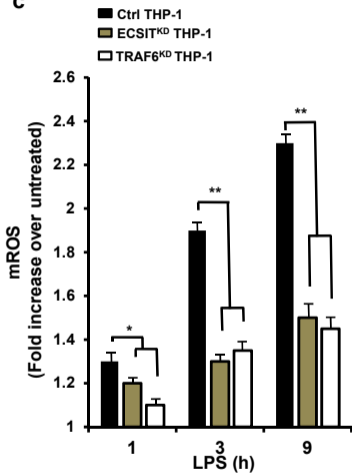

**Supplementary Figure S2**

Supplement: Supplementary file 3 [file Image_2.pdf]

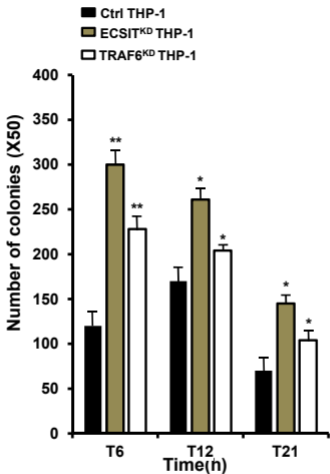

***Supplementary Figure S3***

Supplement: Supplementary file 4 [file Image_3.pdf]

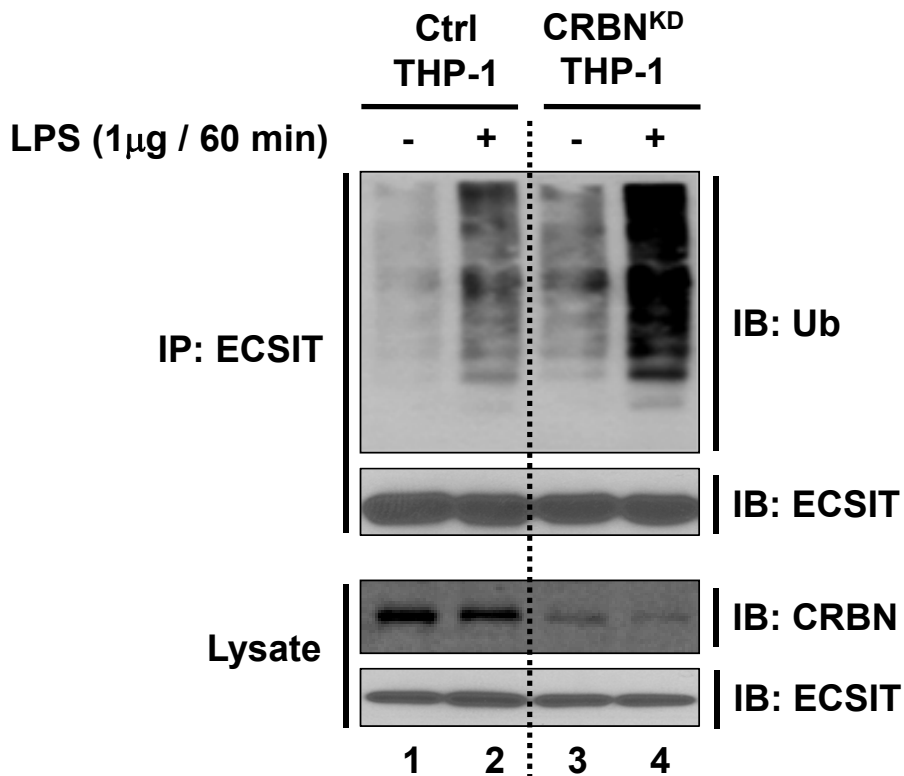

***Supplementary Figure S4***

Supplement: Supplementary file 5 [file Image_4.pdf]

**A**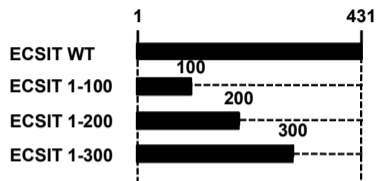**B**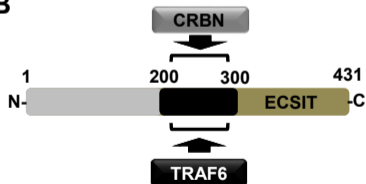**C**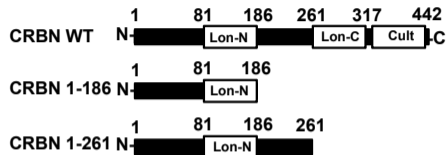**D**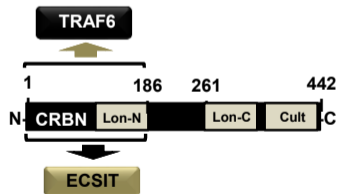

*Supplementary Figure S5*

Supplement: Supplementary file 6 [file Image_5.pdf]

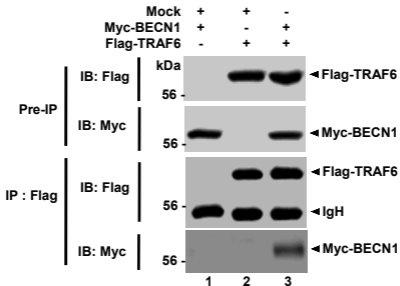

***Supplementary Figure S6***

Supplement: Supplementary file 7 [file Image_6.pdf]
